# Dual-Modified Hyaluronic Acid for Tunable Double Cross-Linked Hydrogel Adhesives

**DOI:** 10.1021/acs.biomac.4c00194

**Published:** 2024-03-08

**Authors:** Cameron Milne, Rijian Song, Melissa Johnson, Chunyu Zhao, Francesca Santoro Ferrer, Sigen A, Jing Lyu, Wenxin Wang

**Affiliations:** † Charles Institute of Dermatology, School of Medicine, University College Dublin, Dublin 4 D04 V1W8, Ireland; ‡ School of Medicine, Anhui University of Science and Technology, Huainan 232001, China; § Research and Clinical Translation Center of Gene Medicine and Tissue Engineering, School of Public Health, Anhui University of Science and Technology, Huainan 232001, China

## Abstract

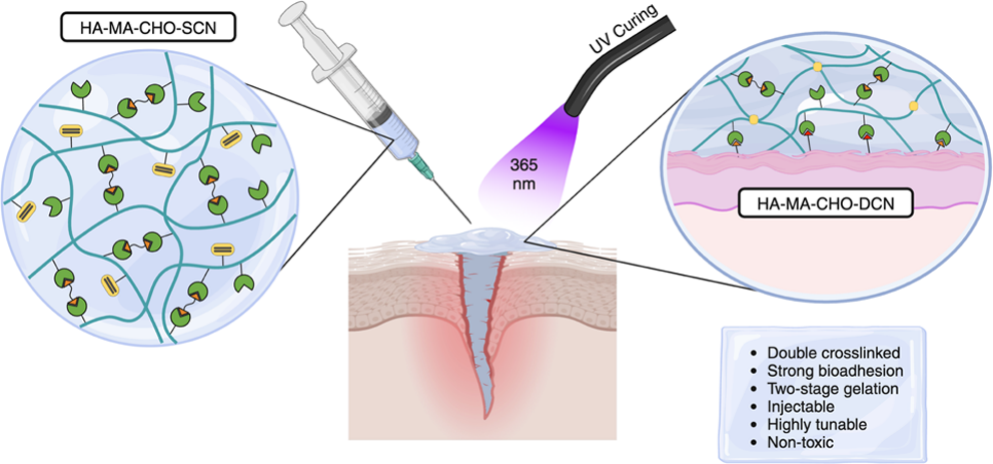

Conventional techniques for the closure of wounds, such as sutures and staples, have significant drawbacks that can negatively impact wound healing. Tissue adhesives have emerged as promising alternatives, but poor adhesion, low mechanical properties, and toxicity have hindered their widespread clinical adoption. In this work, a dual modified, aldehyde and methacrylate hyaluronic acid (HA) biopolymer (HA-MA-CHO) has been synthesized through a simplified route for use as a double cross-linked network (DCN) hydrogel (HA-MA-CHO–DCN) adhesive for the effective closure and sealing of wounds. HA-MA-CHO–DCN cross-links in two stages: initial cross-linking of the aldehyde functionality (CHO) of HA-MA-CHO using a disulfide-containing cross-linker, 3,3′-dithiobis (propionic hydrazide) (DTPH), leading to the formation of a self-healing injectable gel, followed by further cross-linking via ultraviolet (UV) initiated polymerization of the methacrylate (MA) functionality. This hydrogel adhesive shows a stable swelling behavior and remarkable versatility as the storage modulus (*G*′) has shown to be highly tunable (10^3^–10^5^ Pa) for application to many different wound environments. The new HA-MA-CHO–DCN hydrogel showed excellent adhesive properties by surpassing the burst pressure and lap-shear strength for the widely used bovine serum albumin-glutaraldehyde (BSAG) glue while maintaining excellent cell viability.

## 1 Introduction

Following traumatic lacerations and surgical incisions, staples and sutures are the primary means for stopping bleeding, fluid egress, and achieving wound closure.^1−3^ Although these materials are endowed with high tensile strength, they are precluded by being time-consuming to administer, causing additional damage to the wound and increasing the risk of bacterial infiltration and subsequent infection.^4^ Tissue adhesives have shown great promise in their application in wound closure and healing due to their minimally invasive application procedure, ability to enhance wound repair, and the protective seal formed over the wound.^5^ However, existing tissue adhesive formulations have critical limitations that are affecting their translation and efficacy, such as low mechanical strength, weak tissue adhesion, and toxicity.^6^ Materials used for the preparation of tissue adhesives can be classified into two groups: synthetic and bioderived adhesives. The most common synthetic adhesives are cyanoacrylate-based materials, and although they can possess high adhesion strength, they are famed for their lack of biocompatibility, exothermic polymerization reaction, and slow degradation, which can lead to inhibited wound healing.^7,8^ Bioderived Fibrin-based glues, used in clinics today, have good biocompatibility but are not suitable for many applications as they have poor adhesion strength to tissue, low mechanical strength, and also carry a viral contamination risk.^9^ Chemically modified biopolymer hydrogels have been considered a compelling alternative to conventional tissue adhesives as they bring together the best of synthetic and bioderived materials with their mechanical property tunability and structural and biological similarities to the extracellular matrix (ECM).^10^ An optimal tissue adhesive must mimic the mechanical properties of the target tissue in addition to having appropriate mechanical strength, facile and affordable preparation, and biological compatibility. A high mechanical disparity between the tissue and the adhesive can cause mechanical stress at the adhesion interface, which can lead to adhesive failure. Additionally, as is commonly the case with cyanoacrylate-based adhesives, an overly rigid adhesive can inhibit tissue movement and growth.^8,11^ The design of the tissue adhesive system should carefully consider the target tissue’s elastic modulus (*G*′). The *G*′ of biological tissue ranges from ∼1 to ∼100 kPa and so a highly tunable system allows for easier matching of mechanical properties between the tissue and the adhesive.^8^ Injectability, which refers to the ability to temporarily fluidize under sheer stress and then recover to the original mechanical properties postinjection, has also shown to be a feature of great interest for the minimally invasive application of tissue adhesive hydrogels.^12,13^

Hyaluronic acid (HA) is one of the major constituents of the ECM and is an ideal starting block for tissue adhesives due to its biocompatibility and unique structural properties.^14−19^ HA is highly hydrophilic, has anti-inflammatory properties, and can bind to cell receptors making it an ideal material for hydrogel-based tissue adhesives for enhancing wound healing and improved patient outcomes.^20−22^ HA contains multiple sites for chemical modification: the hydroxyl (1 and 2°), carboxylic acid, and *N*-acetyl groups, which allows for additional functionality to be added to the HA structure.^10,23^

The two most common methods to incorporate adhesive properties into a hydrogel system can be divided based on their adhesive mechanisms. (1) A system that creates mechanical interlocks and physical entanglements with the tissue surface; these materials are often cross-linked by free radical polymerization.^24,25^ (2) The formational of covalent bonds between adhesive and tissue, through modification of the adhesive with a chemical binding site, wherein catechol and aldehyde modifications are the most prevalent methods.^14,16,26−28^ However, catechol functionality is susceptible to oxidation, which poses challenges to adhesive performance.^29^ Recently, biopolymer-based hydrogels with aldehyde modification have been successfully developed and demonstrated excellent adhesive performance.^30−34^ Sigen et al. designed an adhesive and injectable hydrogel composed of aldehyde-modified HA and a disulfide cross-linker 3,3′-dithiobis (propionic hydrazide) (DTPH), which showed excellent adhesive capabilities by outperforming bovine serum albumin-glutaraldehyde (BSAG) glue by 65.8% during a lap-shear study with enhanced biocompatibility.^35^ However, the use of aldehyde-modified biopolymers for enhanced adhesive properties often leads to a conflict between injectability and stability.^36^ These systems often rely solely on dynamic Schiff’s base cross-linking for hydrogel preparation, which may help with injectability but often leads to quick gel degradation and low stability.^37^ Recently, sophisticated two-stage cross-linking mechanisms have been developed that may address the challenge of balancing injectability and stability in hydrogels by incorporating a secondary cross-linking step, postinjection, that enhances mechanical strength and resistance to degradation.^34,38−41^

**Figure 1:**
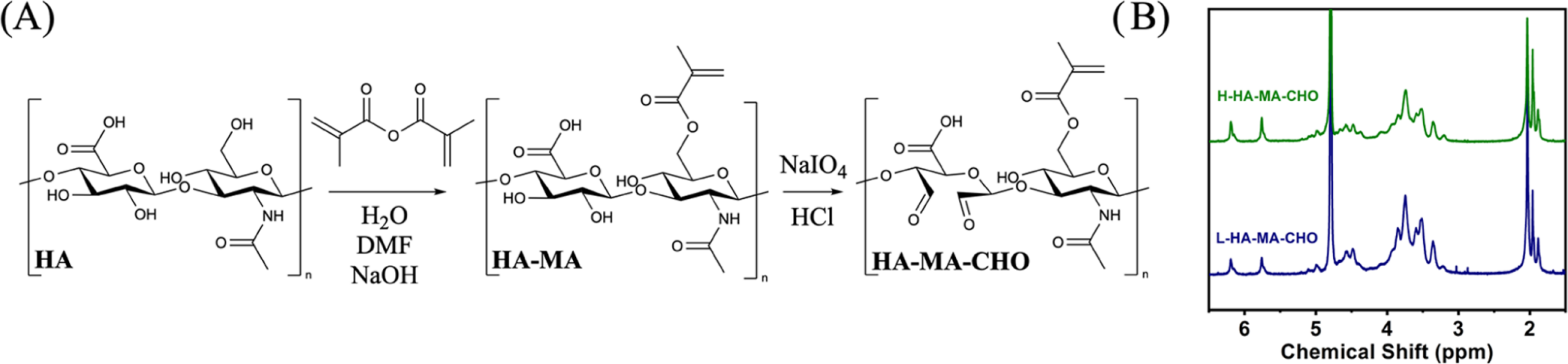
(A) Reaction scheme for one-pot synthesis of HA-MA-CHO. (B) ^1^H NMR spectra for L-HA-MA-CHO and H-HA-MA-CHO in D_2_O.

**Figure 2:**
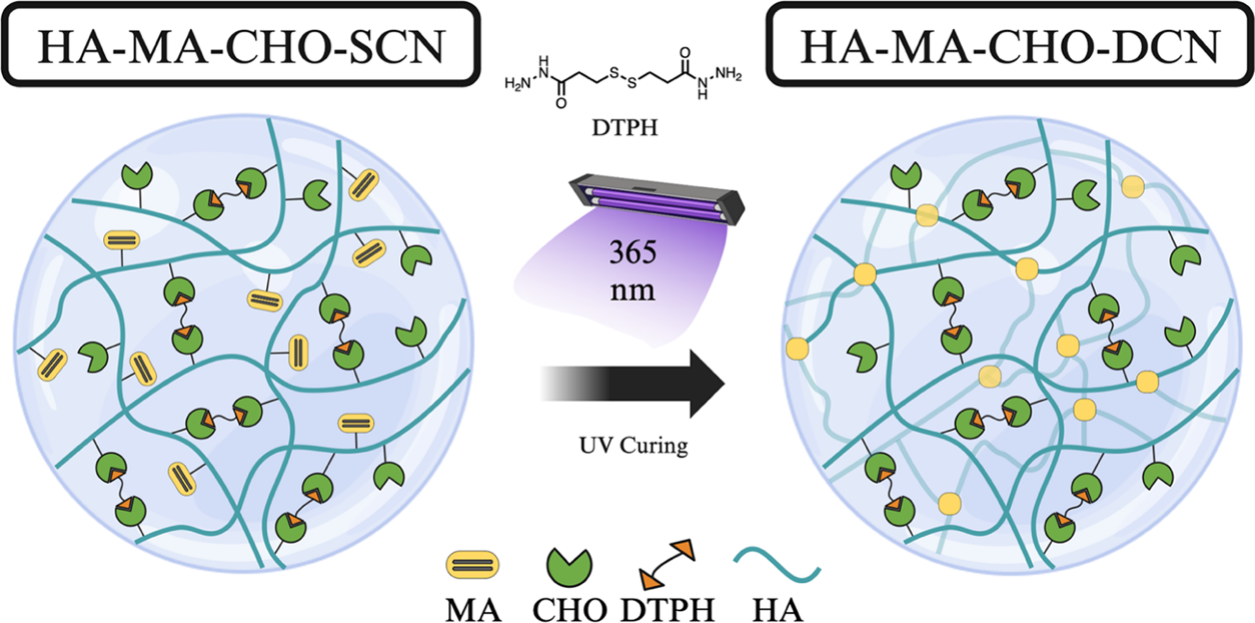
Single cross-linked network HA-MA-CHO with DTPH Schiff’s base cross-linking and UV photopolymerization mechanism to form double cross-linked network HA-MA-CHO. Created with BioRender.com.

In this study, we have developed a facile one-pot synthesis method for aldehyde (CHO) and methacrylate (MA) dual modified hyaluronic acid (HA-MA-CHO) with easily adjustable degrees of MA substitution (MA-DS) and only one step of purification (Figure 1A). This new biopolymer was used to develop a novel double cross-linked HA-MA-CHO hydrogel adhesive with self-healing and highly tunable properties that gelates in two stages. The tunable nature of this material allows for convenient adjustment of the hydrogel mechanical properties for matching the mechanical properties between the wounded tissue and the adhesive. Initially, the network is partially cross-linked by reversible covalent Schiff’s base chemistry between aldehyde (HA-MA-CHO) and DTPH-hydrazide groups (Figure 2), which forms a soft hydrogel (*G*′ approximately 500 Pa) with shear-thinning and self-healing properties. This hydrogel can be injected in situ on the tissue surface through a gel–sol–gel transition where the injected gel thins under shear forces while being pushed through the needle and then, due to the self-healing properties of the Schiff’s base cross-linking, quickly recovers to a gel postinjection. This soft single cross-linked hydrogel has enhanced retention at the wound site when compared to a conventional pre-gel solution with low viscosity.^42^ (2) The secondary cross-linking occurs via the free radical ultraviolet (UV) polymerization of the vinyl groups present in the MA moiety. Using Irgacure 2959, a frequently used and biocompatible photoinitiator, the soft gel can be cured and significantly strengthened in situ.^43−45^ The introduction of the MA cross-linking presents a significant advancement in this hydrogel system, offering expedited and precise curing, enhanced mechanical properties, and improved adhesive strength through mechanical interlocking without compromise in biocompatibility. This, along with the covalent adhesive interactions through the aldehyde functionality, can lead to a synergistic effect and an improved therapeutic approach.

## 2 Materials and Methods

### 2.1 Materials

Sodium hyaluronate (HA, 220 kDa, cosmetic grade) was purchased from Bloomage Freda Biopharm Co. Ltd. 2-Hydroxy-4′-(2-hydroxyethoxy)-2-methylpropiophenone (Irgacure I2959), lithium phenyl-2,4,6-trimethylbenzoylphosphinate (LAP), sodium periodate (NaIO_4_), sodium hydroxide (NaOH), hydrochloric acid (HCl, 37%), trichloroacetic acid (98%), phosphate buffer saline (PBS), hyaluronidase (hyase type II, 300 units/mg), 3,3′-dithiodipropionic acid, sulfuric acid (H_2_SO_4_, 97.5%), hydrazine hydrate (N_2_H_4_·H_2_O, 50–60%), *t*-butyl carbazate (98%), trinitrobenzenesulfonic acid solution (TNBS, 5% (w/v) in H_2_O), dimethylformamide (DMF), dimethylsufoxide-*d*_6_ (DSMO, 99.8 atom % D), and deuterium oxide (D_2_O, 99.9 atom % D) were purchased from Merck, Germany. Ethyl alcohol (99.9%), diethyl ether (99.5%), alamarBlue reagent, and methacrylic anhydride (MA, 94%) were purchased from Fisher Scientific. The LIVE/DEAD viability cytotoxicity kit was purchased from BioScience, Cambridge. Dialysis tubing (MW cut off 8 kDa) was purchased from Spectrum Lab, Ireland. Human keratinocyte cells (HaCaT) and normal human dermal fibroblasts (NHDFs) were purchased from ATCC. Dulbecco’s modified Eagle’s medium (DMEM), fetal bovine serum (FBS), and penicillin/streptomycin (P/S) were purchased from Invitrogen. Full cell culture media for HaCaT cells was prepared using 10% FBS and 1% P/S in DMEM. All chemicals were used as delivered unless noted. All aqueous solutions were prepared with deionized water.

### 2.2 Equipment

^1^H NMR data was obtained at room temperature (298 K) on a Varian VnmrS 400 MHz spectrometer to confirm compound structures in solution and to calculate degrees of substitution of methacrylate on the HA backbone. D_2_O and DMSO-*d*_6_ were used as deuterated solvents. Rheological assessments were carried out at room temperature (298 K) on a TA Instruments HR-2 rheometer equipped with an 8 mm steel parallel plate and a UV light source (OmniCure S1000, Lumen Dynamics Group Inc.). For the lap-shear tests, the steel parallel-plate geometry modulus was replaced with a dynamic mechanical analysis (DMA) modulus. A spectrophotometer (SpectraMax M3Molecular Devices) was used for alamarBlue cell viability and TNBS Assay for determining the quantity of free amines. A Leica DM2500 fluorescence microscope was used to view cell staining.

### 2.3 Synthesis of 3,3′-Dithiobis (Propanoic Hydrazide) (DTPH)

DTPH was synthesized according to a previously published protocol.^46^ Briefly, 3,3′-dithiodipropionic acid (20 g), absolute ethyl alcohol (200 mL), and sulfuric acid (1 drop) were added into a round-bottom flask equipped with a condenser, and the system was heated to reflux overnight until the raw material was fully consumed. The ethyl alcohol was removed by a rotary evaporator, and diethyl ether (300 mL) was added to dissolve the crude oil. The organic layer was washed by H_2_O (3 × 200 mL), then diethyl ether was removed by rotavapor to afford the crude diester (22.5 g, 89.3% yield) as a colorless oil, and the diester was used without any purification. Diester (20 g) and hydrazine hydrate (8 equiv) were separately dissolved into ethyl alcohol (50 mL). The diester solution was added into the solution of hydrazine hydrate dropwise at room temperature (RT). The reaction was heated to 50 °C and monitored by thin-layer chromatography (TLC) until the diester spot had disappeared, and the solution was cooled to RT. DTPH was precipitated and filtered, followed by washing with cool hexane to afford the white crystal. The final product was dried in a vacuum oven for 2 days to fully remove the hydrazine hydrate (15.8 g, 88.1% yield). ^1^H NMR (400 MHz, DMSO-*d*_6_): δ = 9.07 (s, 2H), δ = 4.24 (s, 4H), δ = 2.89 (t, 4H), δ = 2.41 (t, 4H).

### 2.4 One-Pot Synthesis of Dual Modified Aldehyde and Methacrylate Hyaluronic Acid (HA-MA-CHO)

HA (1.00 g, 2.50 mmol) was completely dissolved in ultrapure water (50 mL) at 4 °C. DMF (50 mL) was added to the reaction mixture as a cosolvent. Once the DMF was completely immiscible, the pH of the reaction solution was adjusted to 8–9 with NaOH (5 M). Methacrylic anhydride (1.02 mL, 6.88 mmol or 1.30 mL, 8.75 mmol) was added dropwise while maintaining the pH between 8–9 with NaOH (5 M), and the pH was maintained for 8 h, and then the reaction was left overnight. The pH of the reaction solution was adjusted to 6–7 using HCl (1 M) at RT. Sodium periodate (0.53 g, 2.50 mmol) was added, and the reaction vessel was protected from light. The reaction solution was stirred overnight and then dialyzed against deionized water for 3 days with 4 water changes per day. The purified solution was then flash-frozen and lyophilized to afford a white foam.

### 2.5 ^1^H NMR Calculation of Degree of Methacrylate Substitution (DS)

The structure of HA-MA-CHO was validated by the ^1^H NMR spectrum (Figure S1). Due to the overlapping of the methacrylate −CH_3_ and HA–CH_3_ singlet peaks (B and C) at ∼2.00 ppm, it is not possible to achieve an accurate DS by simply comparing the integration values of these peaks. This method normalizes the integration value for one of the methacrylate vinyl protons to 1 at 5.76 ppm. This will give methacrylate −CH_3_ an integration of 3. The DS may be calculated by comparing the total integration value of the methacrylate −CH_3_ and HA–CH_3_ peaks (*E* = *B* + *C*) to the methacrylate −CH_3_ peak at an integration of 3. i.e.,-What percentage of the overall integration value of the −CH_3_ peaks, is the methacrylate −CH_3_ peak accounting for. The following calculation can be used to calculate the DS %
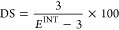


### 2.6 Calculation of the Degree of Oxidation by the Carbazate Trinitrobenzenesulfonate (CTNBS) Assay

A modified trinitrobenzenesulfonate assay was performed to calculate the aldehyde content on the HA backbone (Figure S2). Excess *tert*-butyl carbazate (*t*-bC) (30 mM) was used to react with the aldehyde group in the HA backbone to form a carbazone structure. The excess residual carbazate can be quantified using the TNBS assay, and the aldehyde content is back-calculated based on the total *t*-bC added. Standard samples (5–30 mM) were prepared using *t*-bC. Then, 30 μL of HA-MA-CHO (0.6% w/v) was mixed with 30 μL of *t*-BC (30 mM) in trichloroacetic acid (1% w/v) at RT. Twenty-four h later, TNBS solution (0.6 mL, 6 mM in 0.1 M borate buffer) was added to react with the unreacted *t*-BC for 1 h. Afterward, 200 μL of the reaction solution was diluted with hydrochloric acid (400 μL, 0.5 M), and 150 μL of the sample was added to a 96-well reader plate. Absorbance was measured at 340 nm, and all samples were conducted in triplicate.

### 2.7 Preparation of Tunable Double Cross-Linked HA-MA-CHO and DTPH Hydrogels

Photoinitiator Irgacure 2959 (I2959) 0.5, 0.1, and 0.05% (w/v) and LAP 0.5 % (w/v) were used as the diluent for the HA-MA-CHO and DTPH solutions. HA-MA-CHO–DCN hydrogels were prepared by first mixing HA-MA-CHO 2% with DTP 2% (w/v) at differing HA: DTP volume ratios (25:1–75:1) to form a single cross-linked network (HA-MA-CHO-SCN). The gels were then further cross-linked by UV light (365 nm) or visible light (405 nm) for 60 s.

### 2.8 Rheological Assessment of HA-MA-CHO

Rheological assessments were carried out at room temperature (298 K) on a TA Instruments HR-2 rheometer equipped with an 8 mm steel parallel plate. For time-sweep tests, the HA-MA-CHO-SCN gels were premixed and applied (150 μL) to the plate. The plate gap was then set to 2000 μm, and the test started immediately. HA-MA-CHO-SCN gels were allowed 10 min to cross-link on the plate. The gels were then cured by UV light for 60 s to form HA-MA-CHO–DCN, and then time-sweep data was collected for a further 2 min. The tests were carried out at 25 °C at a frequency of 1.0 Hz and a strain of 1%. Each test was conducted in triplicate. For photoinitiated double cross-linked gels, a curing plate and a UV light (365 nm) or visible light (405) nm source were used (OmniCure S1000, Lumen Dynamics Group Inc.).

### 2.9 Degradation Profile Studies of HA-MA-CHO–DCN Hydrogels

The degradation profile of HA-MA-CHO–DCN double cross-linked hydrogels was determined by measurement of hydrogel weight after incubation in 1× PBS medium (with and without hyaluronidase) in a shaker at 37 °C/100 rpm. 200 μL of HA-MA-CHO-SCN premixture was added to 20 mL glass vials, and the vials were kept at an ∼45° angle to form half-moon-shaped gels. The gels were left to cross-link for 24 h while protected from light to achieve maximum cross-linking density. The gels were then exposed to UV light for 60 s to form HA-MA-CHO–DCN. 5 mL of medium was added to each vial, and the vials were added to the shaker. The degradation profile was then measured by removing the medium completely at the time points and weighing the vials containing the gels. Each experiment was conducted in triplicate, and single cross-linked (without UV) hydrogels were used as a control. The quantity of degradation was calculated by using the following equation
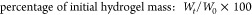
where *W_t_* is the weight of the hydrogel at the scheduled time point and *W*_0_ is the initial weight of the hydrogel. Degradation medium solutions: 5 mL of 1× PBS buffer, 5 mL of 10 U/mL hyaluronidase in 1× PBS buffer, and 5 mL of 100 U/mL hyaluronidase in 1× PBS buffer. Each test was conducted in triplicate.

### 2.10 Adhesive Strength Measurements by the Lap-Shear Experiment

The tissue adhesive strength was measured by the lap-shear test based on ASTM F2255–05 (2015) with slight modification. Glass slides (40 × 12 mm^2^) were coated in a gelatin solution (20% v/w) and dried for use as the substrates. A bovine serum albumin-glutaraldehyde (BSAG) glue composted of bovine serum albumin (BSA, 25% w/v) and glutaraldehyde (10% w/v) were selected as the control group. 72.12 μL of HA-MA-CHO solution 2% (w/v) and 2.88 μL of DTPH solution 2% (w/v) (HA: DTPH volume ratios of 25:1) or 37.5 μL of BSA solution (25% w/v) and 37.5 μL of glutaraldehyde solution (10% w/v) were fully mixed in an Eppendorf and 50 μL was added to one side of the substrate. Another substrate was then covered with an overlapping area of 12 × 5 mm^2^. The HA-MA-CHO-SCN gels were left to cross-link for 30 min. The HA-MA-CHO-SCN gels were then exposed to UV light for 60 s, forming HA-MA-CHO–DCN. 100 g of weight was used to press the cross-linked substrates for 24 h. The lap-shear test was conducted at a constant rate of 5 mm/min, and the lap-shear stress was calculated by the force (*N*) vs the overlap area of the specimen in square meters. L-HA-MA-CHO-SCN gels were included for reference. Each test was conducted in triplicate.

### 2.11 Burst Pressure Studies

The burst pressure was measured based on the standard ASTM F2392–04 method. A circular section (radius = 2.5 cm) of porcine skin was cut and washed in PBS, and the excess fat was removed. A 3 mm hole was punched in the center of the porcine skin. 75 μL of pre-gel solutions was pipetted over and into the 3 mm incision. A bovine serum albumin-glutaraldehyde (BSAG) glue composted of bovine serum albumin (BSA, 25% w/v) and glutaraldehyde (10% w/v) were selected as the control group. HA-MA-CHO-SCN samples were allowed to adhere and gel to the hole for 10 min and then exposed to UV light for 60 s where required. The burst pressure was measured after 20 min of adhesion time to allow for gel stability. The burst pressures were determined by clamping the porcine skin sample over a water inlet that was attached to a pump and manometer and applying force to the sealed hole via water pressure. The pressure at which the incision ruptured was measured. L-HA-MA-CHO-SCN gels were included for reference. Each test was conducted in triplicate.

### 2.12 Cytocompatibility Studies of L-HA-MA-CHO–DCN Hydrogel, HA-MA-CHO Polymers, and DTPH

A direct *in vitro* cytotoxicity test was performed according to ISO 10993–5. All solutions for cell viability tests were prepared using DMEM buffer and filtered for sterilization with a 0.2 μm pore size filter. For the blank control groups, cells were cultured in DMEM buffer without treatment. Cytotoxicity of H-HA-MA-CHO, L-HA-MA-CHO, and DTPH was carried out using HaCaT cell lines. 7.0 × 10^3^ cells/well were seeded into a 96-well plate and cultured overnight in full cell media (Dulbecco’s modified Eagle’s medium (DMEM) with 10% fetal bovine serum (FBS) and 1% penicillin) under standard cell culture conditions (37 °C, 5% CO_2_). The cell medium was then changed to a series of HA-MA-CHO and DTPH solutions (from 0.1 to 1 mg/mL). The cell viability assay was performed after 24 and 72 h following coculture with alamarBlue for 4 h. Cell viability of the HA-MA-CHO and DTPH is calculated based on the untreated cell viability data (blank). LIVE/DEAD kit (calcein/ethidium) staining was utilized to confirm the living status of the cells. After 24 and 72 h, the culture medium was removed and replaced with the LIVE/DEAD stain. After 30 min of incubation at 25 °C, the stain was washed away from the well plates with PBS. The images were captured using a fluorescence microscope. Absorbance values (*n* = 3) are reported as the “relative cell viability,” in which 100% equals the control absorbance. All samples were prepared in triplicate.

### 2.13 Cytocompatibility Studies of the L-HA-MA-CHO–DCN Hydrogel

A direct in vitro cytotoxicity test was performed according to ISO 10993–5. Normal Human Dermal Fibroblasts (NHDFs) were cultured, and the cells were collected by centrifugation before being seeded on the surface of the hydrogels. L-HA-MA-CHO–DCN pre-gel solutions were prepared as above and then filtered by 0.22 μm filter. The L-HA-MA-CHO–DCN hydrogel was prepared in cell culture plates. The blank control was defined as cells seeded directly onto the cell plate without a hydrogel. After 1 h, the cells were seeded to the surface of the hydrogels at a density of 1.0 × 10^5^/well. The cell viability assay was performed after 24 and 72 h following coculture with alamarBlue for 4 h. Cell viability of L-HA-MA-CHO–DCN is calculated based on the untreated cell viability data (blank). LIVE/DEAD kit (calcein/ethidium) staining was utilized to confirm the living status of the seeded cells. After 24 and 72 h, the culture medium was removed and replaced with the LIVE/DEAD stain. After 30 min of incubation at 25 °C, the stain was washed away from the well plates with PBS. The images were captured using a fluorescence microscope. Absorbance values (*n* = 3) are reported as the “relative cell viability,” in which 100% equals the control absorbance. All samples were prepared in triplicate.

### 2.14 Statistical Analysis

All values are expressed as the mean ± standard deviation (SD). Statistical differences between the two groups were determined using Student’s unpaired *t*-test. A *p*-value <0.05 was considered statistically significant.

## 3 Results and Discussion

### 3.1 Synthesis of HA-MA-CHO

The methacrylate functional group allows for polymer cross-linking via a UV photoinitiated free radical polymerization of the vinyl group. This polymerization reaction gives large increases in the elastic modulus and mechanical strength in a rapid time frame. Oxidation of the diol situated on the d-glucuronic acid in the HA backbone will afford aldehyde-modified HA, HA-CHO. The aldehyde functionality allows cross-linking with other modified biopolymers or cross-linkers and is also a site for adhesion to native proteins and collagens on tissue surfaces (−NH_2_, –SH groups, and noncovalent interactions).^47^ Adhesive functionality can also help retain the hydrogel at the wound site for more efficient healing and a more sustained release of therapeutics.^48−51^ As the methacrylate and aldehyde modifications can be synthesized on different moieties of the HA structure, it is possible to synthesize a dual modified hyaluronic acid that has the benefits of both the MA and CHO modifications: HA-MA-CHO.

Although the synthesis of HA-MA-CHO has been previously reported, those methods are protracted processes that require two complicated stages of synthesis and laborious purification, increasing the risk of HA degradation and using copious resources.^52−54^ Previous methods for HA-MA-CHO preparation would require full purification by dialysis and subsequent lyophilization for the intermediate product HA-MA before repeat dissolution in the reaction solvent for the secondary reaction with the oxidizing agent NaIO_4_, followed by a second stage of purification by dialysis. To our knowledge, the greener one-pot synthesis without the need for midstep purification or the use of ethylene glycol has never been reported before. The one-pot synthesis method uses fewer reagents/solvents and requires just one stage of purification by dialysis, saving time and resources. HA-MA-CHO with tailorable methacrylate degree of substitution (MA-DS) was synthesized through a two-step one-pot approach (Figure 1A). The methacrylate moiety was conjugated to the HA primary alcohol via methacrylic anhydride.^55,56^ Due to the poor solubility of methacrylic anhydride in aqueous conditions, DMF was used as a cosolvent for this reaction. The DMF greatly increases the efficiency of this reaction and allows for finer tuning of the final DS.^57,58^ After 24 h, NaIO_4_ was added to the reaction solution, and the hyaluronic acid underwent a ring-opening oxidation reaction to form dialdehyde-modified HA-MA, HA-MA-CHO. The successful chemical modification can be confirmed by ^1^H NMR and TNBS assay. The peaks at 5.8 and 6.2 ppm on ^1^H NMR confirm the presence of methacrylate vinyl protons conjugated to the HA backbone, as shown in Figure 1B. This reaction’s specific and tunable nature allowed for targetable degrees of methacrylate substitution (MA-DS) by fine-tuning reagent feed ratios. Two differing MA-DS were used for hydrogel preparation and assessment: H-HA-MA-CHO (MA-DS = 55%) and L-HA-MA-CHO (MA-DS = 30%). The relative areas of the vinyl proton peaks to the HA and MA −CH_3_ peaks at 2.0 ppm were used to calculate the DS. The two MA-DSs were chosen for the double cross-linked hydrogel network to allow for comparison between two different hydrogel strengths; however, by tailoring the feed ratio of the methacrylic anhydride, we were able to obtain HA-MA-CHO with MA-DS ranging from 10–100%. The oxidation reaction is confirmed by a TNBS assay, which gave an oxidation degree of 15% for H- and L-HA-MA-CHO.

### 3.2 Hydrogel Preparation and Mechanical Assessment

The dialdehyde functionality of HA-MA-CHO allows for a secondary method of cross-linking to add to the vinyl group photopolymerization. DTPH, a dihydrazide containing disulfide, was used as a cross-linker. The terminal DTPH-hydrazide groups react via Schiff’s base chemistry with the HA aldehyde groups on separate HA repeat units to form a single cross-linked network (HA-MA-CHO-SCN) (Figure 2). Commonly, the precursors for photo cross-linked hydrogels display a liquid-like flow, making retention at the wound site before cross-linking difficult.^41^ HA-MA-CHO-SCN hydrogels are endowed with self-healing and shear-thinning properties due to the dynamic and reversible nature of the Schiff base cross-linking mechanism.

**Figure 3:**
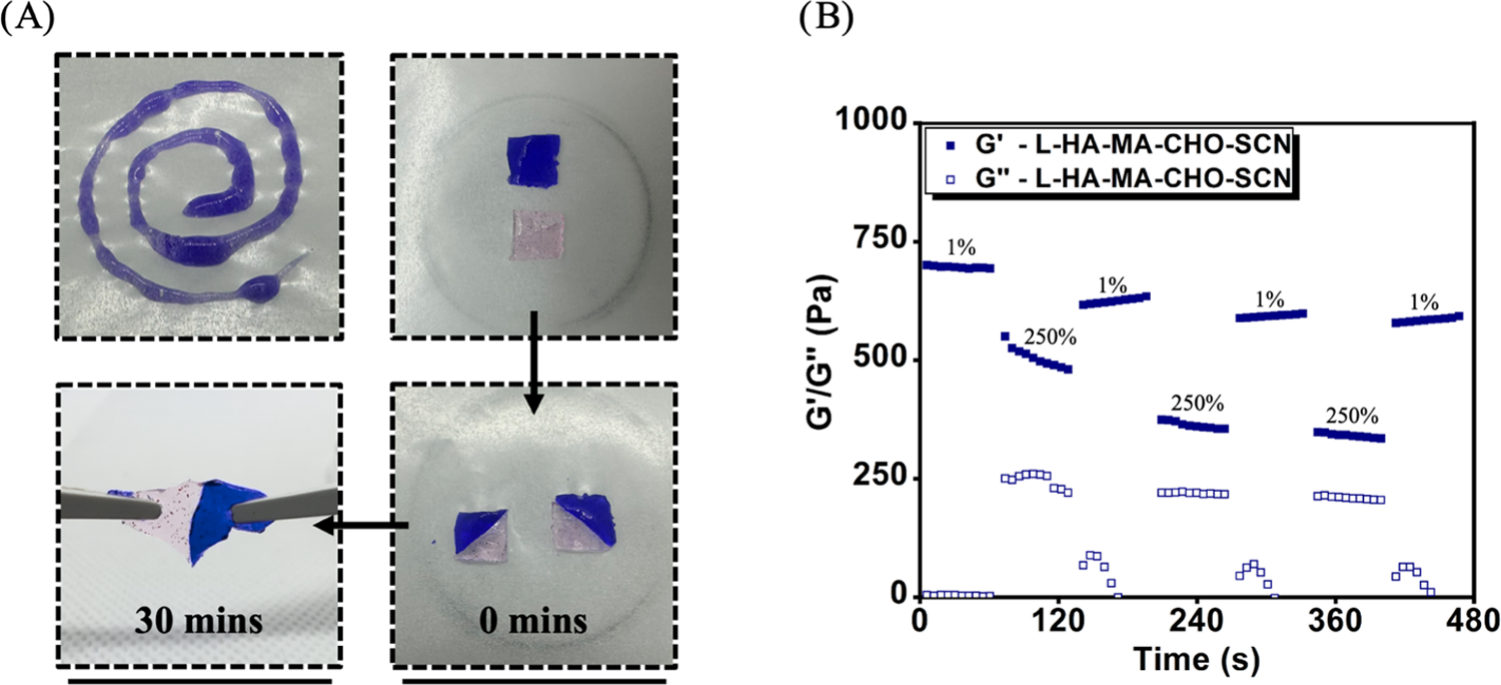
(A) HA-MA-CHO-SCN injected through a 20-gauge needle. The self-healing capability of HA-MA-CHO-SCN over 30 min. (B) Oscillation step-strain test of L-HA-MA-CHO-SCN with an applied strain of 1% (60 s) and 250% (60 s) at 1 Hz.

This allows the SCN hydrogel to be injected through a needle and undergo a gel–sol–gel transition under the shear force while being pushed through the needle tip for simple deposition and improved retention at the wound site. Figure 3A and Video V1 show that the hydrogel may be injected through a 20-gage needle without blocking the needle. To visualize the self-healing ability of SCN-HA-MA-CHO hydrogels, two hydrogels were stained with brilliant blue and methyl red and were cut in half and then stuck to each other. The gels were observed to completely heal in 30 min without external stimulation, showcasing their ability to self-heal (Figure 3A and Video V2). The self-healing ability was also evaluated via an oscillatory step-strain study to confirm recovery of rheological properties after exposure to high strain. Figure 3B shows that *G*′ is recovered quickly after high strain, and the recovery process was repeated for the 3 step-strain cycles. This result confirms the hypothesis that the HA-MA-CHO-SCN system can be used as an injectable hydrogel due to its ability to heal quickly after extrusion. The disulfide bond in the DTPH-HA-MA-CHO cross-link may also aid in the scavenging of ROS and the reduction of oxidative stress in a wound environment.^54,59^ Once in situ, the hydrogel may be cured by exposure to an energy source to produce a HA-MA-CHO double cross-linked hydrogel (HA-MA-CHO–DCN).

In this study, the energy source is UV light (λ = 365 nm), and the photoinitiator used was Irgacure 2959, which was chosen because of its recognized biocompatibility with cells.^60^ If the laboratory/clinic specification does not allow for cross-linking using this wavelength of light, then visible light photoinitiators, such as lithium phenyl-2,4,6-trimethylbenzoylphosphinate (LAP), can be utilized instead (Figure S3).^61^ The photopolymerization of the methacrylate functionality was utilized for the curing of the hydrogel in situ and gave rise to large improvements in mechanical and adhesive properties.

**Figure 4:**
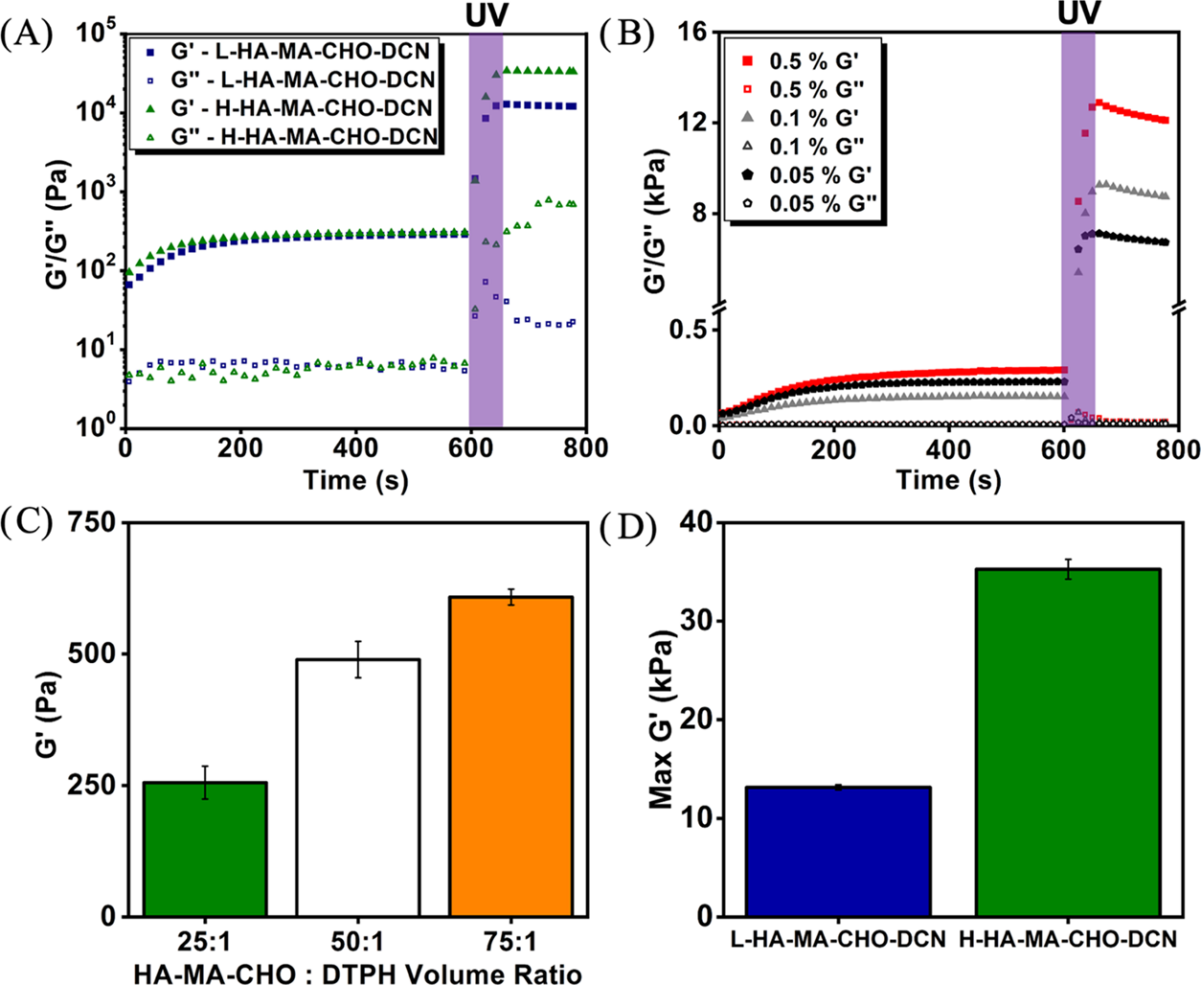
G′/*G*″ assessment was via time-sweep rheology studies at 1 Hz and 1% strain. Gels were exposed to UV light (60 s) after 10 min. (A) H&L HA-MA-CHO–DCN (2% w/v HA-MA-CHO) & DTPH (2% w/v). (B) L-HA-MA-CHO–DCN (2% w/v HA-MA-CHO) with different I2959 photoinitiator concentrations. (C) Effect of L-HA-MA-CHO: DTPH volume ratio on *G*′ of L-HA-MA-CHO-SCN. (D) Max *G*′ H&L HA-MA-CHO–DCN. Data is presented as the average ± standard deviation (*n* = 3).

The gelation process and mechanical properties of HA-MA-CHO–DCN were evaluated in detail via rheological assessment. Time-sweep studies were conducted over 13 min (Figure 4A). A 0.5% Irgacure 2959 solution was used to dissolve the HA-MA-CHO and DTPH. The HA-MA-CHO and DTPH solutions were mixed at a volume ratio of 25:1, the pre-gel solution was injected onto the rheometer plate, and the test was started immediately. The initial increase in storage modulus *G*′ is due to the Schiff’s base reaction between the hydrazide groups on DTPH and the aldehyde groups on HA-MA-CHO; this forms a single cross-linked network HA-MA-CHO-SCN soft hydrogel (*G*′ Approx. 500 Pa). After 300 s, the HA-MA-CHO-SCN has fully cross-linked, and the *G*′ plateaus. At 600 s, the SCN was subjected to UV light (365 nm) for a total irradiation time of 60 s. The storage modulus of both the H&L HA-MA-CHO–DCN was increased due to photoinitiated free radical polymerization of the methacrylate moieties cross-linking the HA backbones. The *G*′ increase was proportional to the DS of the HA-MA-CHO.

The DCN L-HA-MA-CHO and H-HA-MA-CHO hydrogel’s *G*′ increased to 12.9 and 34.1 kPa, respectively. As a wound healing tissue adhesive’s mechanical property should match that of the tissue to which they are administered as closely as possible, adjusting the DS of HA-MA-CHO, and in turn, the *G*′ final DCN hydrogel, is an effective way of applying this hydrogel to different wound types over the body. The effect of using different concentrations of I2959 for the DCN L-HA-MA-CHO hydrogels was also evaluated. Figure 4B shows that decreasing the concentration of the photoinitiator affects the *G*′ of the DCN hydrogel; this is another tool that can be used to fine-tune the final *G*′ of the hydrogel. The *G*′ of the SCN can also be tuned by modifying the HA-MA-CHO to DTPH volume ratios. Figure 4C shows the effects of changing the HA-MA-CHO: DTPH volume ratios from 25:1, 50:1, and 75:1. The rheological testing concluded that by changing the MA-DS, HA-MA-CHO:DTPH volume ratio or photoinitiator concentration, the same hydrogel system (HA-MA-CHO–DCN) can present a wide range of storage moduli. It must be noted that the increases in *G*′ for the SCN are due to the Schiff base bond formation; this reaction consumes two aldehyde groups for every cross-link and, therefore, will affect the adhesive properties of the final hydrogel. For the remainder of this study, all assessments were completed in a 25:1 volume ratio.

### 3.3 Cell Viability of HA-MA-CHO–DCN Hydrogels

**Figure 5:**
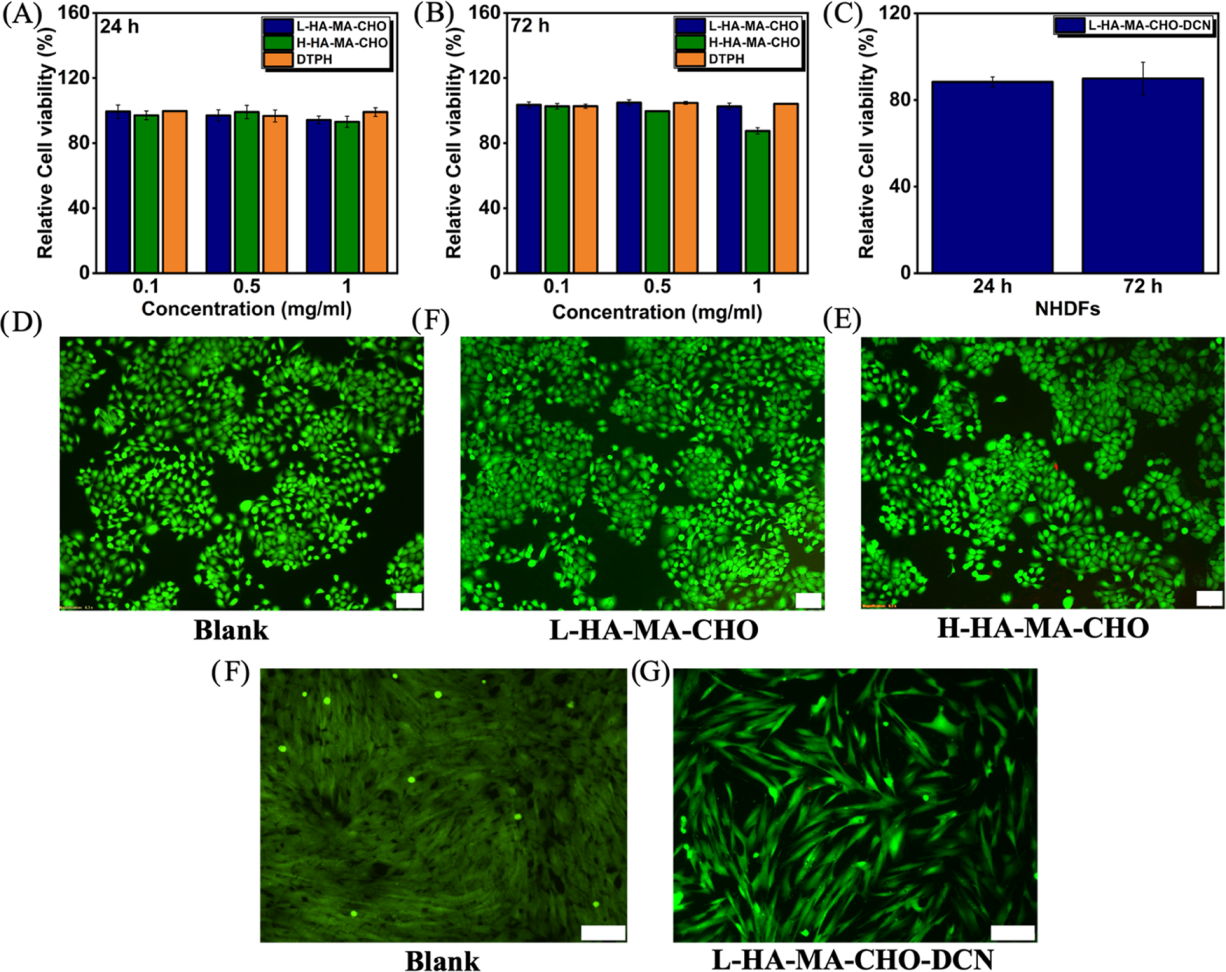
Cytocompatibility of HA-MA-CHO hydrogel components H-HA-MA-CHO, L-HA-MA-CHO DTPH. After 24 (A) and 72 h (B). Cytocompatibility of L-HA-MA-CHO–DCN hydrogels after 24 and 72 h (C). LIVE/DEAD staining images HaCaT cells cocultured with (D) blank, (E) L-HA-MA-CHO, and (F) H-HA-MA-CHO for 72 h. LIVE/DEAD staining images NHDF cells seeded onto the surface of (D) blank and (F) L-HA-MA-CHO–DCN hydrogel for 72 h. Scale Bar = 100 μm. Data is presented as the average ± standard deviation (*n* = 3).

To exhibit the biocompatibility of the HA-MA-CHO and DTPH used in the HA-MA-CHO–DCN hydrogel networks, alamarBlue assays and LIVE/DEAD staining were conducted using human epidermal keratinocyte cells (HaCaTs). The cytotoxicity of the hydrogel materials was evaluated at concentrations of 100, 500, and 1000 μg/mL after cells were cultured for 24 and 72 h. As seen in Figure 5A,B, the results show that there is the reduction of viability was less than 30% for all of the materials tested. According to ISO 10993–5:2009, this indicates that there is no significant cytotoxicity for H&L—HA-MA-CHO and DTPH over both time periods, showing the good biocompatibility of the raw materials. The biocompatibility of L-HA-MA-CHO–DCN hydrogel was investigated by seeding normal human dermal fibroblasts (NHDFs) onto the hydrogel surface; the cell viability was evaluated by alamarBlue assay and imaged by LIVE/DEAD staining at 24 and 72 h. The cells maintained high viability (Figure 5C) and healthy fibroblast spindle morphology (Figure 5G) after exposure to the L-HA-MA-CHO–DCN hydrogel, indicating good biocompatibility of the system.

### 3.4 Degradation Studies

**Figure 6:**
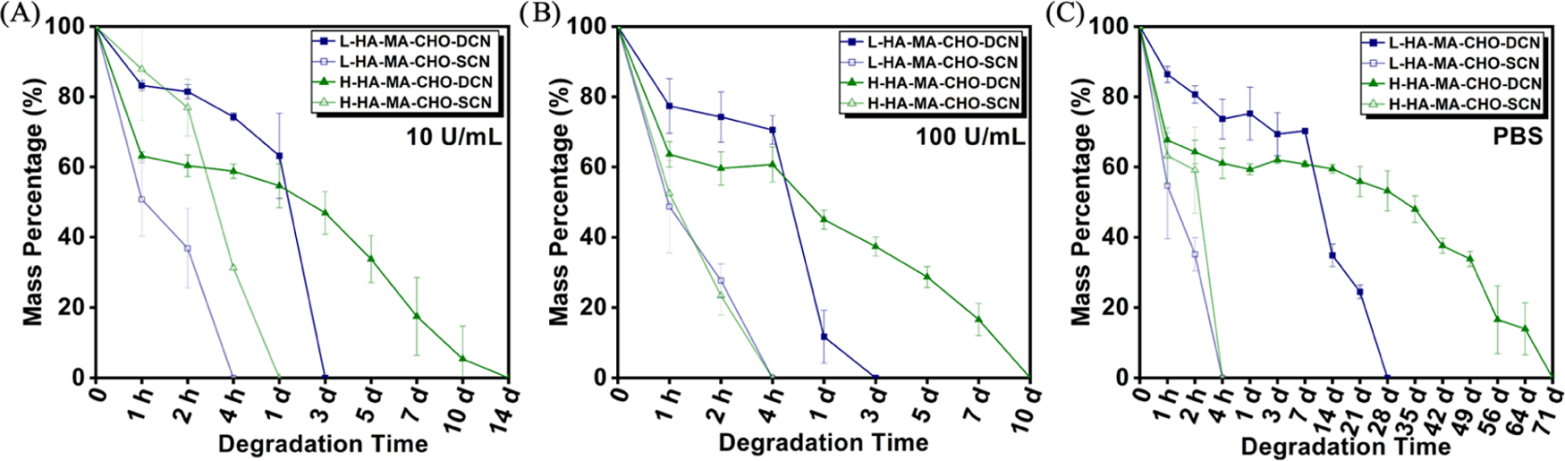
Degradation profiles of L&H HA-MA-CHO hydrogels in (A) 10 U/mL Hyase, (B) 100 U/mL Hyase, and (C) PBS. Data is presented as the average ± standard deviation (*n* = 3).

The degradation profiles for L&H, HA-MA-CHO–DCN, and SCN hydrogels were determined in three separate media, PBS (pH 7.4) and hyaluronidase at concentrations of 10 and 100 U/mL. The degradation rate of the hydrogel adhesive should be designed in relation to the rate of repair of the tissue. If the hydrogel degrades too quickly, it can compromise the integrity of the tissue, leading to potential rupture and inadequate healing. If the degradation is too slow, the adhesive may persist in the wound for an extended period, potentially causing inflammation and interfering with the natural healing processes.^1^ As shown in Figure 6, the DCN hydrogels showed, as expected, an inverse proportionality between MA-DS of the HA-MA-CHO and degradation rate in all media with L-HA-MA-CHO degrading more quickly than H-HA-MA-CHO due to the lower cross-linking density of MA groups. Notably, the L&H HA-MA-CHO–DCN hydrogels have no significant swelling in all media due to their high cross-linking degree. The HA-MA-CHO-SCN degraded significantly more quickly than the DCN hydrogels, which highlights the mechanical stability enhancements gained through the addition of the methacrylate cross-linking. The findings from the hyaluronidase-regulated degradation profile (Figure 6A,B) demonstrate a clear relationship between the concentration of the enzyme and the degradation rate. As hyaluronidase caused an increase in the rate of degradation, it is stipulated that the backbone structure of the HA is well preserved after the one-pot modification reaction. Our results indicate that by adjusting the concentration of hyaluronidase and MA-DS of the HA-MA-CHO, the hydrogel’s degradation can be effectively controlled.

### 3.5 Adhesive Property Assessment

**Figure 7:**
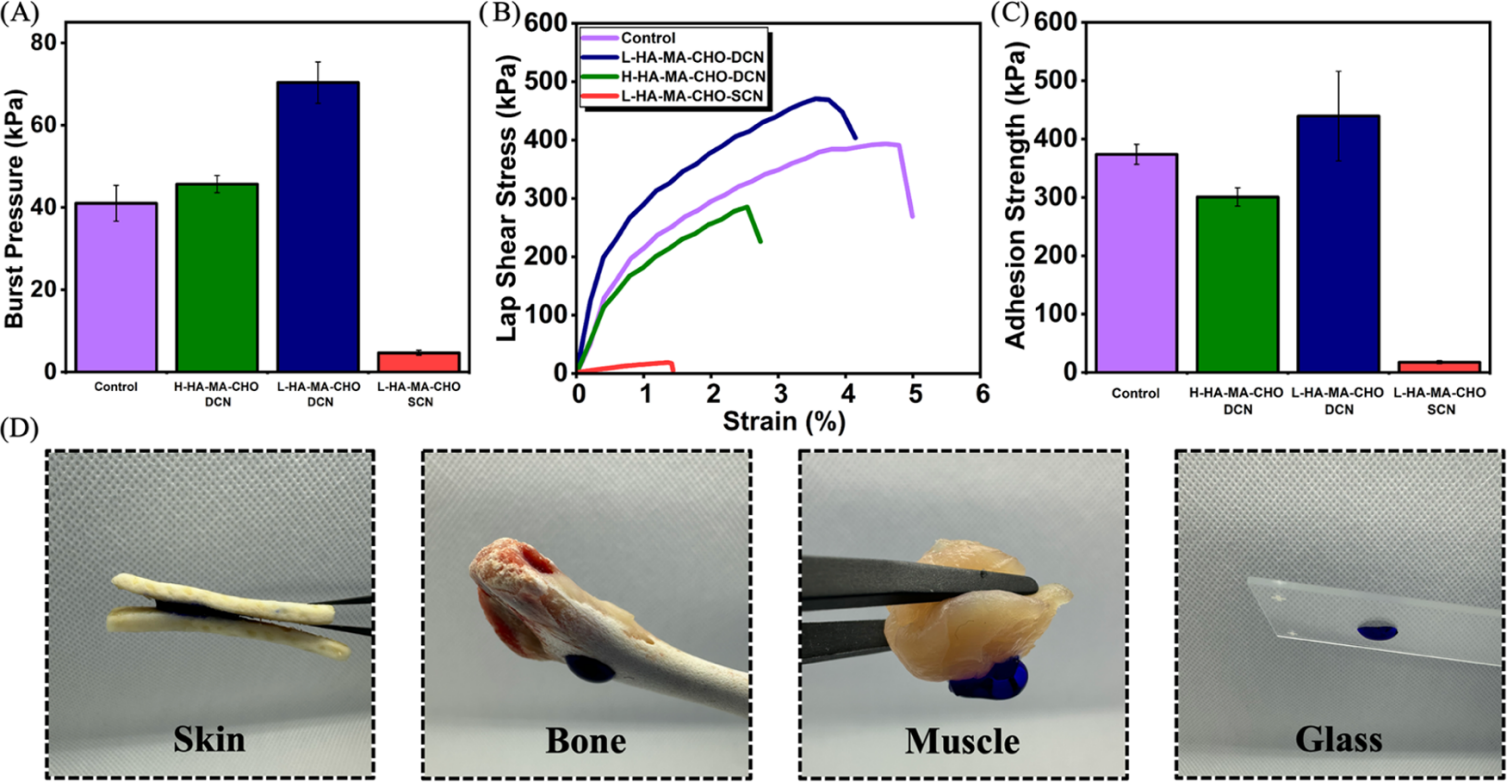
(A) Burst pressure of ex vivo porcine skin with 3 mm incision sealed with HA-MA-CHO hydrogels and control (B) stress–strain curve of lap-shear test of the HA-MA-CHO hydrogels and control. (C) Adhesive strength of HA-MA-CHO hydrogels and control with a minimum of 5 samples per group. (D) L-HA-MA-CHO–DCN adhesion to a variety of surfaces, including skin, bone, muscle, and glass. Data is presented as average ± standard deviation (*n* = 3).

To quantitatively evaluate the adhesive performance of the HA-MA-CHO–DCN hydrogels, we completed lap-shear and burst pressure assessments were completed. Bovine serum albumin-glutaraldehyde (BSAG) glue hydrogels were used as the control as they are widely used in surgery today.^62^ L-HA-MA-CHO-SCN hydrogels were tested for reference. The burst pressure procedure followed an adapted version of the standard ASTM F2392–04 method, Figure 7A shows the burst pressure of L&H HA-MA-CHO–DCN hydrogels, L-HA-MA-CHO-SCN, and control hydrogel sealants. The L-HA-MA-CHO–DCN hydrogels were able to withstand 1.5 times the pressure of the commercially available control sealant (70.3 and 45.7 kPa). Literature suggests sealant hydrogels should exhibit a burst pressure of 27 kPa for arterial vascular applications, 9 kPa for corneal incision sealants, 30 kPa for thoracic aorta applications, and 10 kPa for tracheal applications.^63^ Since both HA-MA-CHO–DCN hydrogels exhibited a higher burst pressure than the control sealant and met the requirements for a diverse range of human tissues, it can be concluded that the system is suitable for application in many human tissues.

The lap-shear strength of HA-MA-CHO–DCN and control hydrogels was evaluated following a procedure based on the ASTM F2255–05. Figure 7B shows the lap-shear stress–strain curve, and Figure 7C shows the shear adhesive strength of HA-MA-CHO–DCN/SCN and control hydrogels. The L-HA-MA-CHO–DCN hydrogels showed greater adhesive strength compared to the control (439.3 and 300.9 kPa). However, the control hydrogel was able to withstand a higher strain before adhesive failure. This is believed to be due to the high cross-linking density of the HA-MA-CHO–DCN hydrogels. This endows the hydrogels with a brittle characteristic and makes them susceptible to cohesive failure. Although H-HA-MA-CHO performed similarly to the control sealant, it was not able to withstand as high pressure or shear strength as the L-HA-MA-CHO. Again, this could be attributed to the higher cross-linking density in H-HA-MA-CHO–DCN compared to L-HA-MA-CHO–DCN, and while the H-HA-MA-CHO–DCN hydrogel has a higher storage modulus, it is less able to withstand deformation. In this study, the adhesion strength from HA-MA-CHO–DCN is significantly higher than those found in commercially available fibrin-based glues (2–40 kPa),^53^ dopamine conjugated HA hydrogels (∼200 kPa),^64^ or commercially available cyanoacrylate glues (∼80 kPa).^65^ These results reinforce the fact that the HA-MA-CHO–DCN hydrogels are a promising alternative to commercially recognized tissue adhesives and sealants. The HA-MA-CHO–DCN hydrogels outperform the commercially available control sealant in both tests while also retaining the inherent biocompatibility and biofunctions of hyaluronic acid.

## 4 Conclusions

The one-pot synthesis of tunable dual modified hyaluronic acid (HA-MA-CHO) has been successfully developed. HA-MA-CHO was then used to create a double cross-linked hydrogel adhesive (HA-MA-CHO–DCN). The two-stage gelation process, involving initial cross-linking through Schiff’s base chemistry and subsequent strengthening through UV-polymerization, provides a versatile and easily applicable solution for wound sealing. The hydrogel network was endowed with aldehyde and vinyl functionality, which provided enhanced adhesive performance when compared to the BSA/glutaraldehyde control adhesive system. The HA-MA-CHO–DCN system also showed excellent biocompatibility. It can be expected that HA-MA-CHO–DCN hydrogels will be very appealing in the field of biomedical science and can be used to address the limitations of current tissue adhesives.

## Supporting Information Available

^1^H NMR spectra for HA-MA-CHO; standard curve of *t*-BC absorbance for the TNBS assay, aldehyde content and oxidation degree of HA-MA-CHOs; and time-sweep rheology study of H&L HA-MA-CHO–DCN (2% w/v HA-MA-CHO) & DTPH (2% w/v) with LAP photoinitiator (Figures S1–S3) (PDF)
Injectability of HA-MA-CHO-SCN (Video V1) (AVI)
Self-adhesive properties of HA-MA-CHO-SCN (Video V2) (AVI)

## Supplementary Material

bm4c00194_si_001.pdf

bm4c00194_si_002.avi

bm4c00194_si_003.avi

## References

[ref1] Muffly T. M.; Tizzano A. P.; Walters M. D. The History and Evolution of Sutures in Pelvic Surgery. J. R. Soc. Med. 2011, 104 (3), 107–112. 10.1258/jrsm.2010.100243. 21357979 PMC3046193

[ref2] Mackenzie D. The History of Sutures. Med. Hist. 1973, 17 (2), 158–168. 10.1017/S0025727300018469. 4578411 PMC1081445

[ref3] O’Rorke R. D.; Pokholenko O.; Gao F.; Cheng T.; Shah A.; Mogal V.; Steele T. W. J. Addressing Unmet Clinical Needs with UV Bioadhesives. Biomacromolecules 2017, 18 (3), 674–682. 10.1021/acs.biomac.6b01743. 28124557

[ref4] Tajirian A. L.; Goldberg D. J. A Review of Sutures and Other Skin Closure Materials. J. Cosmet. Laser Ther. 2010, 12 (6), 296–302. 10.3109/14764172.2010.538413. 21142740

[ref5] Khanlari S.; Dubé M. A. Bioadhesives: A Review. Macromol. React. Eng. 2013, 7 (11), 573–587. 10.1002/mren.201300114.

[ref6] Chen T.; Chen Y.; Rehman H. U.; Chen Z.; Yang Z.; Wang M.; Li H.; Liu H. Ultratough, Self-Healing, and Tissue-Adhesive Hydrogel for Wound Dressing. ACS Appl. Mater. Interfaces 2018, 10 (39), 33523–33531. 10.1021/acsami.8b10064. 30204399

[ref7] Taboada G. M.; Yang K.; Pereira M. J. N.; Liu S. S.; Hu Y.; Karp J. M.; Artzi N.; Lee Y. Overcoming the Translational Barriers of Tissue Adhesives. Nat. Rev. Mater. 2020, 5 (4), 310–329. 10.1038/s41578-019-0171-7.

[ref8] Nam S.; Mooney D. Polymeric Tissue Adhesives. Chem. Rev. 2021, 121 (18), 11336–11384. 10.1021/acs.chemrev.0c00798. 33507740

[ref9] Bouten P. J. M.; Zonjee M.; Bender J.; Yauw S. T. K.; Van Goor H.; Van Hest J. C. M.; Hoogenboom R. The Chemistry of Tissue Adhesive Materials. Prog. Polym. Sci. 2014, 39 (7), 1375–1405. 10.1016/j.progpolymsci.2014.02.001.

[ref10] Burdick J. A.; Prestwich G. D. Hyaluronic Acid Hydrogels for Biomedical Applications. Adv. Mater. 2011, 23 (12), H41–H56. 10.1002/adma.201003963. 21394792 PMC3730855

[ref11] Chu W.; Nie M.; Ke X.; Luo J.; Li J. Recent Advances in Injectable Dual Crosslinking Hydrogels for Biomedical Applications. Macromol. Biosci. 2021, 21 (7), 2100109 10.1002/mabi.202100109. 33908175

[ref12] Guvendiren M.; Lu H. D.; Burdick J. A. Shear-Thinning Hydrogels for Biomedical Applications. Soft Matter 2012, 8 (2), 260–272. 10.1039/C1SM06513K.

[ref13] Bertsch P.; Diba M.; Mooney D. J.; Leeuwenburgh S. C. G. Self-Healing Injectable Hydrogels for Tissue Regeneration. Chem. Rev. 2023, 123 (2), 834–873. 10.1021/acs.chemrev.2c00179. 35930422 PMC9881015

[ref14] Zhou D.; Li S.; Pei M.; Yang H.; Gu S.; Tao Y.; Ye D.; Zhou Y.; Xu W.; Xiao P. Dopamine-Modified Hyaluronic Acid Hydrogel Adhesives with Fast-Forming and High Tissue Adhesion. ACS Appl. Mater. Interfaces 2020, 12 (16), 18225–18234. 10.1021/acsami.9b22120. 32227982

[ref15] Zhang Y.; Zheng Y.; Shu F.; Zhou R.; Bao B.; Xiao S.; Li K.; Lin Q.; Zhu L.; Xia Z. In Situ-Formed Adhesive Hyaluronic Acid Hydrogel with Prolonged Amnion-Derived Conditioned Medium Release for Diabetic Wound Repair. Carbohydr. Polym. 2022, 276, 118752 10.1016/j.carbpol.2021.118752. 34823781

[ref16] Fang Y.; Zhang L.; Chen Y.; Wu S.; Weng Y.; Liu H. Polysaccharides Based Rapid Self-Crosslinking and Wet Tissue Adhesive Hemostatic Powders for Effective Hemostasis. Carbohydr. Polym. 2023, 312, 120819 10.1016/j.carbpol.2023.120819. 37059547

[ref17] Nguyen D. D.; Yao C.-H.; Luo L.-J.; Chen H.-C.; Hsueh Y.-J.; Ma D. H.-K.; Lai J.-Y. Oxidation-Mediated Scaffold Engineering of Hyaluronic Acid-Based Microcarriers Enhances Corneal Stromal Regeneration. Carbohydr. Polym. 2022, 292, 119668 10.1016/j.carbpol.2022.119668. 35725168

[ref18] Li M.; Shi X.; Yang B.; Qin J.; Han X.; Peng W.; He Y.; Mao H.; Kong D.; Gu Z. Single-Component Hyaluronic Acid Hydrogel Adhesive Based on Phenylboronic Ester Bonds for Hemostasis and Wound Closure. Carbohydr. Polym. 2022, 296, 119953 10.1016/j.carbpol.2022.119953. 36087997

[ref19] Bermejo-Velasco D.; Kadekar S.; da Costa M. V. T.; Oommen O. P.; Gamstedt K.; Hilborn J.; Varghese O. P. First Aldol Cross-Linked Hyaluronic Acid Hydrogel: Fast and Hydrolytically Stable Hydrogel with Tissue Adhesive Properties. ACS Appl. Mater. Interfaces 2019, 11 (41), 38232–38239. 10.1021/acsami.9b10239. 31550878

[ref20] A S.; Zeng M.; Johnson M.; Creagh-Flynn J.; Xu Q.; Tai H.; Wang W. Green Synthetic Approach for Photo-Cross-Linkable Methacryloyl Hyaluronic Acid with a Tailored Substitution Degree. Biomacromolecules 2020, 21 (6), 2229–2235. 10.1021/acs.biomac.0c00196. 32271548

[ref21] Mele E. Electrospinning of Natural Polymers for Advanced Wound Care: Towards Responsive and Adaptive Dressings. J. Mater. Chem. B 2016, 4 (28), 4801–4812. 10.1039/C6TB00804F. 32263137

[ref22] Collins M. N.; Birkinshaw C. Hyaluronic Acid Based Scaffolds for Tissue Engineering—A Review. Carbohydr. Polym. 2013, 92 (2), 1262–1279. 10.1016/j.carbpol.2012.10.028. 23399155

[ref23] Pérez L. A.; Hernández R.; Alonso J. M.; Pérez-González R.; Sáez-Martínez V. Hyaluronic Acid Hydrogels Crosslinked in Physiological Conditions: Synthesis and Biomedical Applications. Biomedicines 2021, 9 (9), 1113 10.3390/biomedicines9091113. 34572298 PMC8466770

[ref24] Chandrasekharan A.; Seong K.-Y.; Yim S.-G.; Kim S.; Seo S.; Yoon J.; Yang S. Y. In Situ Photocrosslinkable Hyaluronic Acid-Based Surgical Glue with Tunable Mechanical Properties and High Adhesive Strength. J. Polym. Sci., Part A: Polym. Chem. 2019, 57 (4), 522–530. 10.1002/pola.29290.

[ref25] Guo Y.; Wang Y.; Zhao X.; Li X.; Wang Q.; Zhong W.; Mequanint K.; Zhan R.; Xing M.; Luo G. Snake Extract–Laden Hemostatic Bioadhesive Gel Cross-Linked by Visible Light. Sci. Adv. 2021, 7 (29), eabf9635 10.1126/sciadv.abf9635. 34261653 PMC8279511

[ref26] Li J.; Celiz A. D.; Yang J.; Yang Q.; Wamala I.; Whyte W.; Seo B. R.; Vasilyev N. V.; Vlassak J. J.; Suo Z.; Mooney D. J. Tough Adhesives for Diverse Wet Surfaces. Science 2017, 357 (6349), 378–381. 10.1126/science.aah6362. 28751604 PMC5905340

[ref27] Zhao P.; Wei K.; Feng Q.; Chen H.; Wong D. S. H.; Chen X.; Wu C.-C.; Bian L. Mussel-Mimetic Hydrogels with Defined Cross-Linkers Achieved via Controlled Catechol Dimerization Exhibiting Tough Adhesion for Wet Biological Tissues. Chem. Commun. 2017, 53 (88), 12000–12003. 10.1039/C7CC07215E. 29052668

[ref28] Zhou F.; Yang Y.; Zhang W.; Liu S.; Shaikh A. B.; Yang L.; Lai Y.; Ouyang H.; Zhu W. Bioinspired, Injectable, Tissue-Adhesive and Antibacterial Hydrogel for Multiple Tissue Regeneration by Minimally Invasive Therapy. Appl. Mater. Today 2022, 26, 101290 10.1016/j.apmt.2021.101290.

[ref29] Pei X.; Wang J.; Cong Y.; Fu J. Recent Progress in Polymer Hydrogel Bioadhesives. J. Polym. Sci. 2021, 59 (13), 1312–1337. 10.1002/pol.20210249.

[ref30] Zhao Y.; Yi B.; Hu J.; Zhang D.; Li G.; Lu Y.; Zhou Q. Double Cross-Linked Biomimetic Hyaluronic Acid-Based Hydrogels with Thermo-Stimulated Self-Contraction and Tissue Adhesiveness for Accelerating Post-Wound Closure and Wound Healing. Adv. Funct. Mater. 2023, 33 (26), 2300710 10.1002/adfm.202300710.

[ref31] Song H.; Xing L.; Liu W.; Wang X.; Hou Z.; Wang Y.; Zhang Z.; Li Y.; Li T.; Wang X.; Chen H.; Xing S.; Xu J. Biomimetic and Multifunctional Hemostatic Hydrogel with Rapid Thermoresponsive Gelation and Robust Wet Adhesion for Emergency Hemostasis: A Rational Design Based on Photo-Cross-Linking Coordinated Hydrophilic–Hydrophobic Balance Strategies. Biomacromolecules 2023, 24 (7), 3327–3344. 10.1021/acs.biomac.3c00357. 37366605

[ref32] Zhang L.; Luo B.; An Z.; Zheng P.; Liu Y.; Zhao H.; Zhang Z.; Gao T.; Cao Y.; Zhang Y.; Pei R. MMP-Responsive Nanoparticle-Loaded, Injectable, Adhesive, Self-Healing Hydrogel Wound Dressing Based on Dynamic Covalent Bonds. Biomacromolecules 2023, 24 (12), 5769–5779. 10.1021/acs.biomac.3c00773. 37950669

[ref33] Tsai C.-C.; Chandel A. K. S.; Mitsuhashi K.; Fujiyabu T.; Inagaki N. F.; Ito T. Injectable, Shear-Thinning, Self-Healing, and Self-Cross-Linkable Benzaldehyde-Conjugated Chitosan Hydrogels as a Tissue Adhesive. Biomacromolecules 2024, 25 (2), 1084–1095. 10.1021/acs.biomac.3c01117. 38289249

[ref34] Yuan M.; Xu S.; Zhou Y.; Chen Y.; Song J.; Ma S.; He Y.; Mao H.; Kong D.; Gu Z. A Facile Bioorthogonal Chemistry-Based Reversible to Irreversible Strategy to Surmount the Dilemma between Injectability and Stability of Hyaluronic Acid Hydrogels. Carbohydr. Polym. 2023, 317, 121103 10.1016/j.carbpol.2023.121103. 37364964

[ref35] A S.; Xu Q.; Johnson M.; Creagh-Flynn J.; Venet M.; Zhou D.; Lara-Sáez I.; Tai H.; Wang W. An Injectable Multi-Responsive Hydrogel as Self-Healable and on-Demand Dissolution Tissue Adhesive. Appl. Mater. Today 2021, 22, 100967 10.1016/j.apmt.2021.100967.

[ref36] Haines-Butterick L.; Rajagopal K.; Branco M.; Salick D.; Rughani R.; Pilarz M.; Lamm M. S.; Pochan D. J.; Schneider J. P. Controlling Hydrogelation Kinetics by Peptide Design for Three-Dimensional Encapsulation and Injectable Delivery of Cells. Proc. Natl. Acad. Sci. U.S.A. 2007, 104 (19), 7791–7796. 10.1073/pnas.0701980104. 17470802 PMC1876526

[ref37] Wang H.; Zhu D.; Paul A.; Cai L.; Enejder A.; Yang F.; Heilshorn S. C. Covalently Adaptable Elastin-Like Protein–Hyaluronic Acid (ELP–HA) Hybrid Hydrogels with Secondary Thermoresponsive Crosslinking for Injectable Stem Cell Delivery. Adv. Funct. Mater. 2017, 27 (28), 1605609 10.1002/adfm.201605609. 33041740 PMC7546546

[ref38] Lu H. D.; Soranno D. E.; Rodell C. B.; Kim I. L.; Burdick J. A. Secondary Photocrosslinking of Injectable Shear-Thinning Dock-and-Lock Hydrogels. Adv. Healthcare Mater. 2013, 2 (7), 1028–1036. 10.1002/adhm.201200343. 23299998

[ref39] Wan T.; Fan P.; Zhang M.; Shi K.; Chen X.; Yang H.; Liu X.; Xu W.; Zhou Y. Multiple Crosslinking Hyaluronic Acid Hydrogels with Improved Strength and 3D Printability. ACS Appl. Bio Mater. 2022, 5 (1), 334–343. 10.1021/acsabm.1c01141. 35014821

[ref40] Lou J.; Liu F.; Lindsay C. D.; Chaudhuri O.; Heilshorn S. C.; Xia Y. Dynamic Hyaluronan Hydrogels with Temporally Modulated High Injectability and Stability Using a Biocompatible Catalyst. Adv. Mater. 2018, 30 (22), 1705215 10.1002/adma.201705215. 29682801

[ref41] Jongprasitkul H.; Parihar V. S.; Turunen S.; Kellomäki M. pH-Responsive Gallol-Functionalized Hyaluronic Acid-Based Tissue Adhesive Hydrogels for Injection and Three-Dimensional Bioprinting. ACS Appl. Mater. Interfaces 2023, 15 (28), 33972–33984. 10.1021/acsami.3c02961. 37409522 PMC10360037

[ref42] Guedes G.; Wang S.; Fontana F.; Figueiredo P.; Lindén J.; Correia A.; Pinto R. J. B.; Hietala S.; Sousa F. L.; Santos H. A. Dual-Crosslinked Dynamic Hydrogel Incorporating Mo154 with pH and NIR Responsiveness for Chemo-Photothermal Therapy. Adv. Mater. 2021, 33 (40), 2007761 10.1002/adma.202007761. PMC11468987 34382257

[ref43] Eren T. N.; Kariksiz N.; Demirci G.; Tuncel D.; Okte N.; Acar H. Y.; Avci D. Irgacure 2959-Functionalized Poly(Ethyleneimine)s as Improved Photoinitiators: Enhanced Water Solubility, Migration Stability and Visible-Light Operation. Polym. Chem. 2021, 12 (18), 2772–2785. 10.1039/D1PY00298H.

[ref44] Mironi-Harpaz I.; Wang D. Y.; Venkatraman S.; Seliktar D. Photopolymerization of Cell-Encapsulating Hydrogels: Crosslinking Efficiency versus Cytotoxicity. Acta Biomater. 2012, 8 (5), 1838–1848. 10.1016/j.actbio.2011.12.034. 22285429

[ref45] Williams C. G.; Malik A. N.; Kim T. K.; Manson P. N.; Elisseeff J. H. Variable Cytocompatibility of Six Cell Lines with Photoinitiators Used for Polymerizing Hydrogels and Cell Encapsulation. Biomaterials 2005, 26 (11), 1211–1218. 10.1016/j.biomaterials.2004.04.024. 15475050

[ref46] Vercruysse K. P.; Marecak D. M.; Marecek J. F.; Prestwich G. D. Synthesis and in Vitro Degradation of New Polyvalent Hydrazide Cross-Linked Hydrogels of Hyaluronic Acid. Bioconjugate Chem. 1997, 8 (5), 686–694. 10.1021/bc9701095. 9327132

[ref47] Zhang L.; Liu M.; Zhang Y.; Pei R. Recent Progress of Highly Adhesive Hydrogels as Wound Dressings. Biomacromolecules 2020, 21 (10), 3966–3983. 10.1021/acs.biomac.0c01069. 32960043

[ref48] Jung H.; Kim M. K.; Lee J. Y.; Choi S. W.; Kim J. Adhesive Hydrogel Patch with Enhanced Strength and Adhesiveness to Skin for Transdermal Drug Delivery. Adv. Funct. Mater. 2020, 30 (42), 2004407 10.1002/adfm.202004407.

[ref49] Chen J.; Wang D.; Wang L.-H.; Liu W.; Chiu A.; Shariati K.; Liu Q.; Wang X.; Zhong Z.; Webb J.; Schwartz R. E.; Bouklas N.; Ma M. An Adhesive Hydrogel with “Load-Sharing” Effect as Tissue Bandages for Drug and Cell Delivery. Adv. Mater. 2020, 32 (43), 2001628 10.1002/adma.202001628. PMC7606513 32945035

[ref50] Deng M.; Wu Y.; Ren Y.; Song H.; Zheng L.; Lin G.; Wen X.; Tao Y.; Kong Q.; Wang Y. Clickable and Smart Drug Delivery Vehicles Accelerate the Healing of Infected Diabetic Wounds. J. Controlled Release 2022, 350, 613–629. 10.1016/j.jconrel.2022.08.053. 36058354

[ref51] He Z.; Luo H.; Wang Z.; Chen D.; Feng Q.; Cao X. Injectable and Tissue Adhesive EGCG-Laden Hyaluronic Acid Hydrogel Depot for Treating Oxidative Stress and Inflammation. Carbohydr. Polym. 2023, 299, 120180 10.1016/j.carbpol.2022.120180. 36876795

[ref52] Patel J. M.; Loebel C.; Saleh K. S.; Wise B. C.; Bonnevie E. D.; Miller L. M.; Carey J. L.; Burdick J. A.; Mauck R. L. Stabilization of Damaged Articular Cartilage with Hydrogel-Mediated Reinforcement and Sealing. Adv. Healthcare Mater. 2021, 10 (10), 2100315 10.1002/adhm.202100315. PMC8224478 33738988

[ref53] Chen J.; Yang J.; Wang L.; Zhang X.; Heng B. C.; Wang D.-A.; Ge Z. Modified Hyaluronic Acid Hydrogels with Chemical Groups That Facilitate Adhesion to Host Tissues Enhance Cartilage Regeneration. Bioact. Mater. 2021, 6 (6), 1689–1698. 10.1016/j.bioactmat.2020.11.020. 33313448 PMC7708943

[ref54] Gao Y.; Li Z.; Huang J.; Zhao M.; Wu J. In Situ Formation of Injectable Hydrogels for Chronic Wound Healing. J. Mater. Chem. B 2020, 8 (38), 8768–8780. 10.1039/D0TB01074J. 33026387

[ref55] Burdick J. A.; Chung C.; Jia X.; Randolph M. A.; Langer R. Controlled Degradation and Mechanical Behavior of Photopolymerized Hyaluronic Acid Networks. Biomacromolecules 2005, 6 (1), 386–391. 10.1021/bm049508a. 15638543 PMC2678566

[ref56] Smeds K. A.; Grinstaff M. W. Photocrosslinkable Polysaccharides Forin Situ Hydrogel Formation. J. Biomed. Mater. Res. 2001, 54 (1), 115–121. 10.1002/1097-4636(200101)54:1<115::AID-JBM14>3.0.CO;2-Q. 11077410

[ref57] Hachet E.; Van Den Berghe H.; Bayma E.; Block M. R.; Auzély-Velty R. Design of Biomimetic Cell-Interactive Substrates Using Hyaluronic Acid Hydrogels with Tunable Mechanical Properties. Biomacromolecules 2012, 13 (6), 1818–1827. 10.1021/bm300324m. 22559074

[ref58] Bencherif S. A.; Srinivasan A.; Horkay F.; Hollinger J. O.; Matyjaszewski K.; Washburn N. R. Influence of the Degree of Methacrylation on Hyaluronic Acid Hydrogels Properties. Biomaterials 2008, 29 (12), 1739–1749. 10.1016/j.biomaterials.2007.11.047. 18234331

[ref59] Xu Q.; Venet M.; Wang W.; Creagh-Flynn J.; Wang X.; Li X.; Gao Y.; Zhou D.; Zeng M.; Lara-Sáez I.; A S.; Tai H.; Wang W. Versatile Hyperbranched Poly(β-Hydrazide Ester) Macromers as Injectable Antioxidative Hydrogels. ACS Appl. Mater. Interfaces 2018, 10 (46), 39494–39504. 10.1021/acsami.8b15006. 30376290

[ref60] Bryant S. J.; Nuttelman C. R.; Anseth K. S. Cytocompatibility of UV and Visible Light Photoinitiating Systems on Cultured NIH/3T3 Fibroblasts in Vitro. J. Biomater. Sci., Polym. Ed. 2000, 11 (5), 439–457. 10.1163/156856200743805. 10896041

[ref61] Maiz-Fernández S.; Pérez-Álvarez L.; Silván U.; Vilas-Vilela J. L.; Lanceros-Mendez S. Photocrosslinkable and Self-Healable Hydrogels of Chitosan and Hyaluronic Acid. Int. J. Biol. Macromol. 2022, 216, 291–302. 10.1016/j.ijbiomac.2022.07.004. 35798076

[ref62] Bures M.; Höffler H.-K.; Friedel G.; Kyriss T.; Boedeker E.; Länger F.; Zardo P.; Zhang R. Albumin-Glutaraldehyde Glue for Repair of Superficial Lung Defect: An in Vitro Experiment. J. Cardiothorac. Surg. 2016, 11 (1), 63 10.1186/s13019-016-0443-x. 27072534 PMC4828862

[ref63] Ghobril C.; Grinstaff M. W. The Chemistry and Engineering of Polymeric Hydrogel Adhesives for Wound Closure: A Tutorial. Chem. Soc. Rev. 2015, 44 (7), 1820–1835. 10.1039/C4CS00332B. 25649260

[ref64] Fan P.; Dong Q.; Yang J.; Chen Y.; Yang H.; Gu S.; Xu W.; Zhou Y. Flexible Dual-Functionalized Hyaluronic Acid Hydrogel Adhesives Formed in Situ for Rapid Hemostasis. Carbohydr. Polym. 2023, 313, 120854 10.1016/j.carbpol.2023.120854. 37182954

[ref65] Lauto A.; Mawad D.; Foster L. J. R. Adhesive Biomaterials for Tissue Reconstruction. J. Chem. Technol. Biotechnol. 2008, 83 (4), 464–472. 10.1002/jctb.1771.

