# Supplementary material for: Dual-Modified Hyaluronic Acid for Tunable Double Cross-Linked Hydrogel Adhesives
Source: Biomacromolecules. 2024 Mar 8;25(4):2645–55. doi: 10.1021/acs.biomac.4c00194 (PMC11005013; doi:10.1021/acs.biomac.4c00194)
Supplement: Supplementary file 1 — bm4c00194_si_001.pdf [file bm4c00194_si_001.pdf]

Supporting Information

Dual-Modified Hyaluronic Acid for Tunable Double Crosslinked Hydrogel Adhesives.

Cameron Milne, Rijian Song, Melissa Johnson, Chunyu Zhao, Francesca Santoro Ferrer, Sigen A*, Jing Lyu and Wenxin Wang**

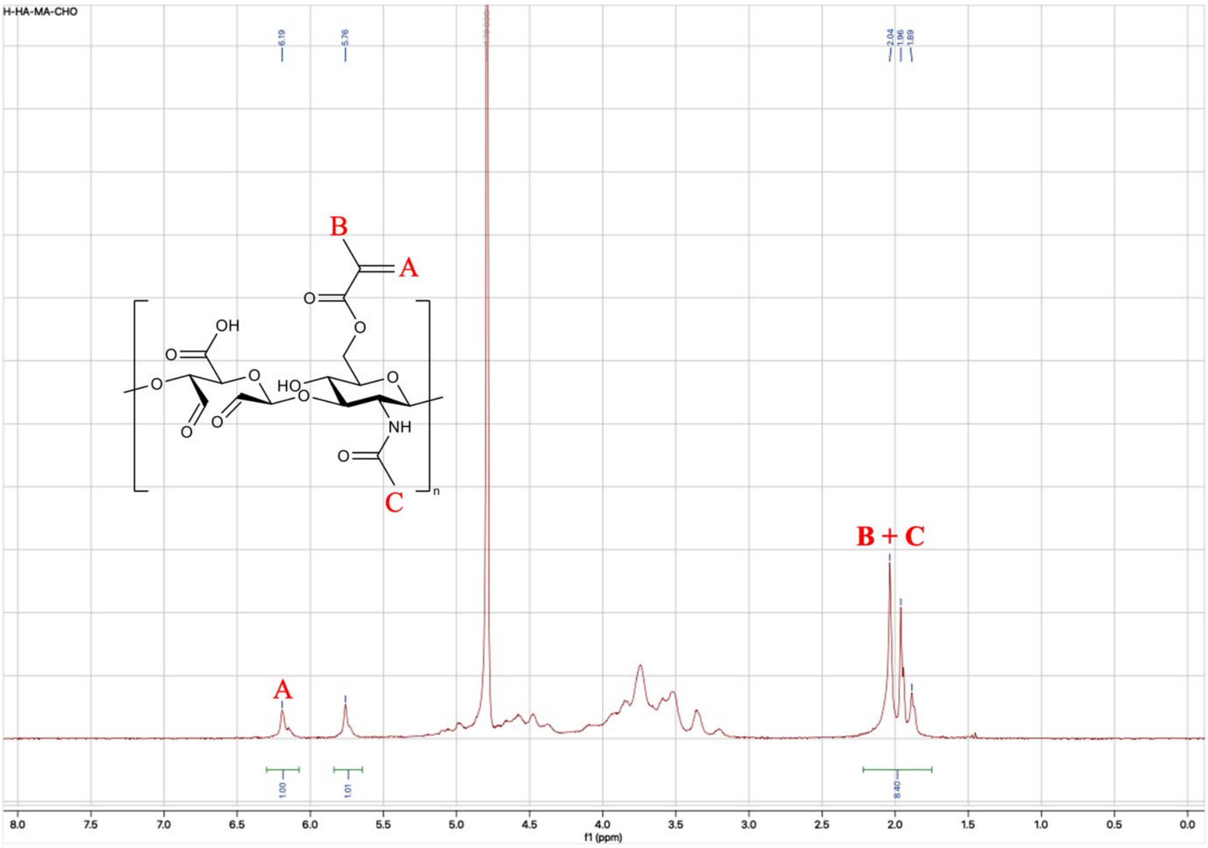

Figure S1. ^{1}H-NMR spectrum of HA-MA-CHO.

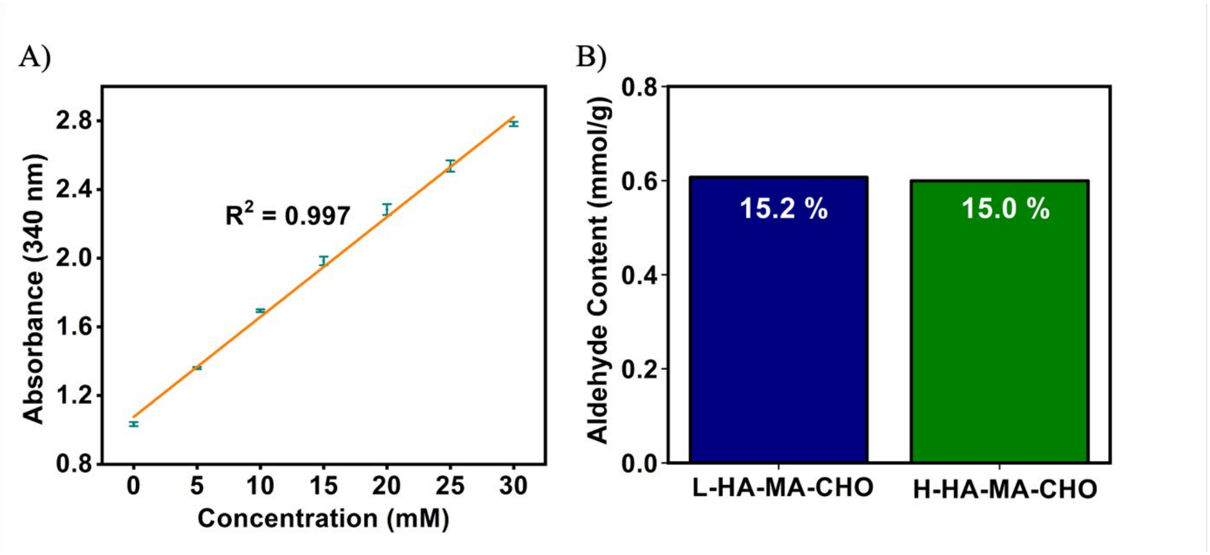

Figure S2. (A) Standard curve of *t*-BC absorbance for TNBS assay. (B) Aldehyde content and oxidation degree of HA-MA-CHOs.

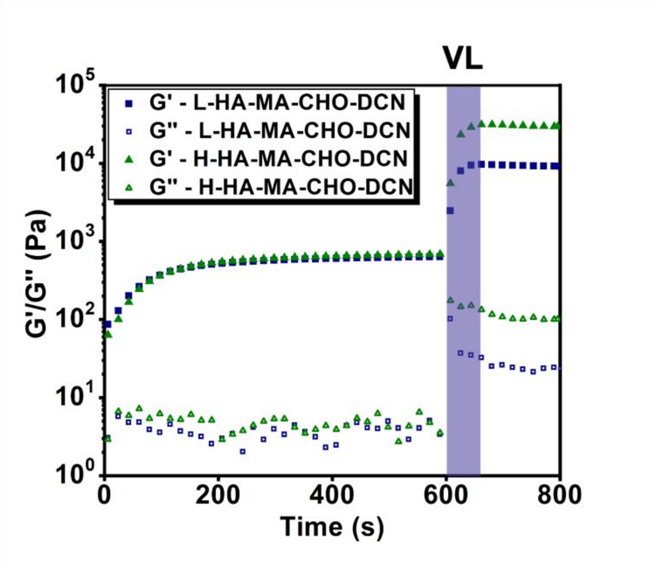

Figure S3 G'/G" assessment via time sweep studies of H & L HA-MA-CHO-DCN (2 % w/v HA-MA-CHO) & DTPH (2 % w/v) with LAP photoinitiator at 1 Hz and 1 % strain. Gels were exposed to visible light (405 nm, 60 s) after 10 mins.

Videos uploaded separately.

Video (**V1**) Injectability of HA-MA-CHO-SCN. (**V2**) Self-adhesive properties of HA-MA-CHO-SCN.
